# Analysis of Obstetrics and Gynecology Residency Program Website Contents in the United States of America

**DOI:** 10.7759/cureus.79065

**Published:** 2025-02-15

**Authors:** Harneet K Cheema, Xinyan Li, Mehr Jain, Faisal Khosa

**Affiliations:** 1 Health Sciences, University of Ottawa, Ottawa, CAN; 2 Krieger School of Arts and Sciences, Johns Hopkins University, Baltimore, USA; 3 Obstetrics and Gynecology, University of Ottawa, Ottawa, CAN; 4 Radiology, Vancouver General Hospital, Vancouver, CAN

**Keywords:** gynecology, obstetrics, residency program, usa, website

## Abstract

Background

Obstetrics and gynecology (OB/GYN) residency program websites are essential resources for applicants when selecting a residency program. Assessing these websites against a 58-point criterion can provide program administrators with actionable insights to enhance their content and make them more informative for applicants.

Objective

This study aims to evaluate the comprehensiveness of content on American OB/GYN residency program websites.

Methods

We reviewed American OB/GYN residency program websites listed on the Fellowship and Residency Electronic Interactive Database (FRIEDA). A 58-point criterion was developed based on the Accreditation Council for Graduate Medical Education (ACGME) common program requirements and prior studies. The criteria included seven categories. Programs without a webpage, military-based programs, and non-US-based programs were excluded.

Results

A total of 272 program websites met the inclusion criteria. On average, websites contained only 28 of the 58 study criteria (48.6%). The most commonly included information was the residency manual (59.1%), while details about didactics and program structure were the least commonly included (40.7%).

Conclusion

The majority of American OB/GYN residency program websites lack comprehensive information for applicants. Residency programs should consider incorporating key details such as application information, introduction to the program, residency manual, didactics and program description, research, current resident information, and graduate/post-residency placement.

## Introduction

In today's digital age, the Internet has become an essential resource for billions of people worldwide [1]. It provides aspiring medical professionals with quick and easy access to crucial information that can guide their educational choices and future career decisions [2]. Program websites play a key role in helping medical applicants select residency and fellowship programs by offering vital information [3]. This is particularly true across all specialties, including the obstetrics and gynecology (OB/GYN) residency programs. 

As residency programs become increasingly competitive [4], websites are essential tools for applicants to better understand programs and identify those that align with their interests. Current literature has examined residency program websites across various specialties, such as the Canadian OB/GYN residency and fellowship programs [5], North American urogynecology fellowship programs [3], Society for Maternal-Fetal Medicine fellowship programs [2], anesthesiology residency programs [6], and psychiatry residency programs [7].

Despite frequent use by applicants [8], research has shown that many residency program websites lack essential information [9,10]. This study aims to evaluate the comprehensiveness of publicly available OB/GYN residency program websites in the United States of America (US) and provide valuable recommendations for improving website content to better serve OB/GYN applicants.

## Materials and methods

Ethics Review Board approval was not required for this study, as data were compiled from publicly available online content. Data collection took place over two months, from February 2020 to March 2020, and was completed by a single reviewer. All 289 US-based OB/GYN residency programs listed on the Fellowship and Residency Electronic Interactive Database (FRIEDA) [11] and the Association of American Medical Colleges (AAMC) [12] were accessed. For this study, only accredited US residency programs listed on FREIDA and AAMC with an official website were included in the data collection. Programs with non-searchable website links through Google, Puerto Rican programs, and military-based programs were excluded. To identify the 272 OB/GYN residency program websites, several searches were conducted. Websites were located through either Google or FREIDA and were verified by manually searching the program identification number on the FREIDA website.

A 58-point criterion was established based on both the Accreditation Council for Graduate Medical Education (ACGME) Common Program Requirements [13] and common criteria used in program website analysis studies in other specialties [14,15]. Each website was evaluated against website analysis criteria consisting of seven categories: application, introduction, residency manual information, education, research, current residents, and alumni (Table 1). A score was awarded for each item if the corresponding information appeared on the official residency program website or subpages of the institution's website. Additionally, all hyperlinks within these websites or subpages were reviewed to ensure a thorough assessment of the criteria. Information not found on these web pages was considered absent. All 58 criteria were evaluated to conduct a comprehensive analysis, consistent with previous studies assessing program websites across various specialties [3,5,15]. Given the limited data on which website elements are most important to applicants, this approach allows for a thorough assessment of website content and provides insight into how programs compare to an ideal standard.

**Table 1:** Website content categories for US obstetrics and gynecology residency programs ERAS: Electronic Residency Application Service, USMLE: United States Medical Licensing Examination.

Website criteria	Websites providing information (N = 272)	Percentage of websites providing information (%)	Average per category (%)
				Application information			50.1
1. Application process	229	84.2	
2. Link to ERAS	169	62.1	
3. Contact email	159	58.5	
4. Mailing address	70	25.7	
5. Selection criteria	211	77.6	
6. Interview process	106	39	
7. Interview dates	96	35.3	
8. Audition or visiting rotations	87	32	
9. International medical students	175	64.3	
10. Minimum USMLE scores	70	25.7	
Introduction			46.2
11. Chairman's message	58	21.3	
12. Program director's message	133	48.9	
13. Chief resident’s message	6	2.2	
14. Department changes and news	67	24.6	
15. Comprehensive faculty listings	232	85.3	
16. Facility description	147	54	
17. Training sites	244	89.7	
Residency manual			59.1
18. Incentives	136	50	
19. Salary	229	84.2	
20. Benefits	238	87.5	
21. Parking	156	57.4	
22. Vacation	227	83.5	
23. Maternal leave mentioned	142	52.2	
24. Paternal leave mentioned	139	51.1	
25. Meal allowance	178	65.4	
26. Moonlighting mentioned	137	50.4	
27. Information about surrounding areas	144	52.9	
28. Socializing events for residents	68	25	
29. Resident wellness	149	54.8	
Didactics/program information			40.7
30. Description of didactics	169	62.1	
31. Journal club	163	59.9	
32. Meetings and conferences	168	61.8	
33. Rotation schedule	200	73.5	
34. Responsibility progression	106	39	
35. Call requirements (night float)	140	51.5	
36. Surgical case/responsibility progression	41	15.1	
37. Surgical statistics	52	19.1	
38. Imaging and procedural numbers	32	11.8	
39. Imaging equipment description	16	5.9	
40. Ultrasound component	192	70.6	
41. Simulation lab	145	53.3	
42. Robotics	107	39.3	
43. International opportunities	59	21.7	
44. Educational resources available to residents	83	30.5	
Research			42.6
45. Research requirements	166	61	
46. Active/past research projects	93	34.2	
47. Research resources	60	22.1	
48. Support to present research	148	54.4	
Current residents' information			48.2
49. Number of residents	238	87.5	
50. Current residents' listings	237	87.1	
51. Residents' photos	217	79.8	
52. Residents' education	207	76.1	
53. Residents' hometown	66	24.3	
54. Residents' research interest	5	1.8	
55. Residents' academic interests	23	8.5	
56. Residents' extracurricular activities	64	23.5	
Graduate/post-residency			43.6
57. Fellowship	121	44.5	
58. Career placement	118	43.4	

Descriptive statistics were used to summarize the mean website scores. Programs were categorized by geographic location: Northeast, Midwest, West, and South [16]. The proportion of information present was calculated for each category, by program, and by geographic location. All data were computed using Microsoft Excel (Microsoft Corp., Redmond, WA, US).

## Results

Of the 289 OB/GYN residency programs, 272 met the inclusion criteria and were analyzed. The program websites included an average of 28 out of 58 (48.6% ± 13%) criteria. Table 1 provides a detailed breakdown of each content category and the percentage of websites that included each criterion. It shows that information related to the residency manual was the most commonly included (59.1%). This category included elements such as incentives, salary, benefits, parking, vacation, parental leave, meal allowance, moonlighting, and residency life. On the other hand, didactics and program information were the least frequently included, with only 40.7% of programs providing this content. This category included details on didactics, journal club, meetings and conferences, and rotation schedules. 

**Figure 1:**
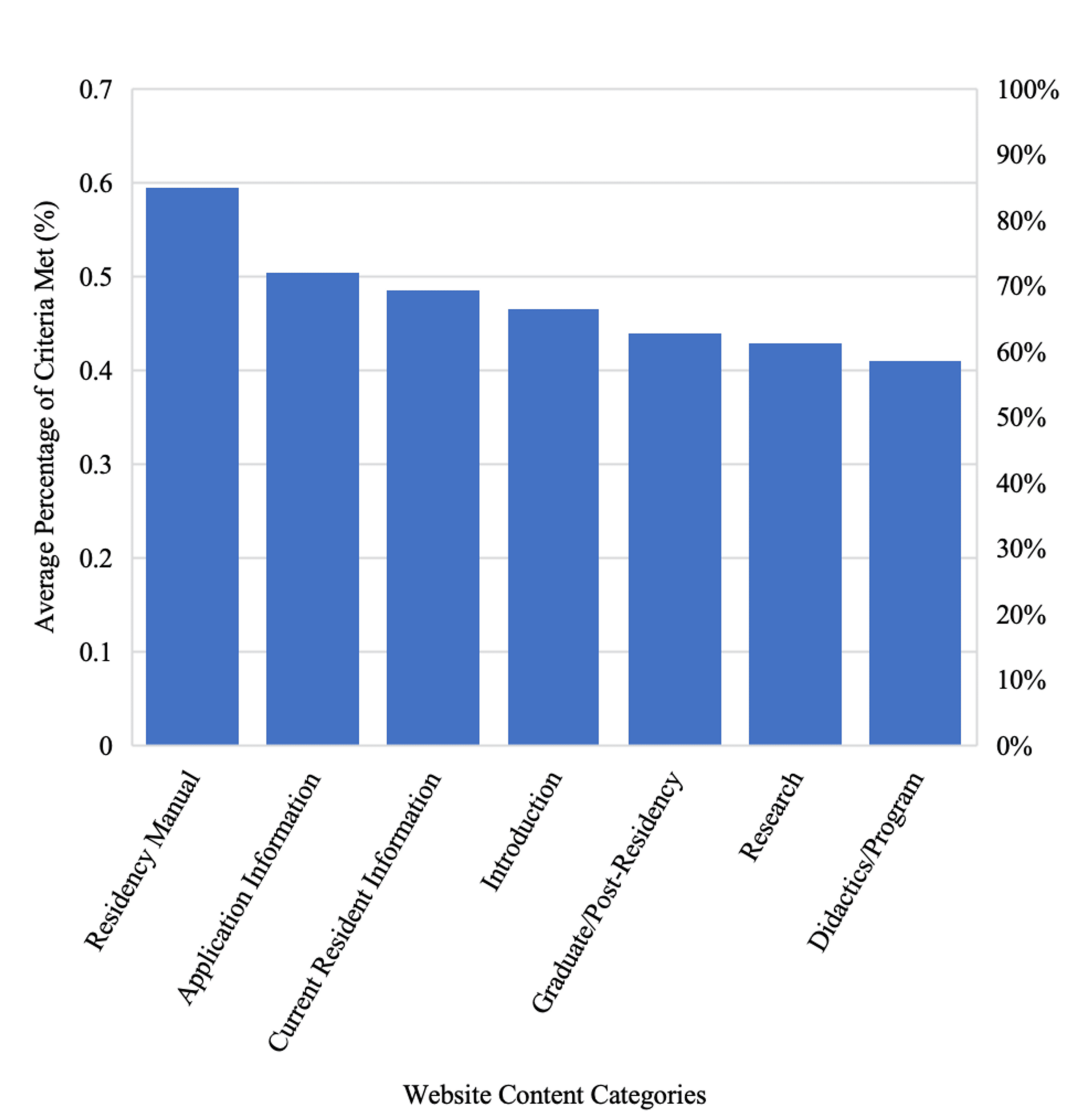
Average percentage of criteria met by website content category for obstetrics and gynecology residency programs in the US

Figure 1 visually represents this variation, demonstrating how information about the residency manual was most frequently included across program websites, while didactics and program information were the least represented.

Of the 272 programs analyzed, only 10 included more than 70% of the 58 criteria on their websites, and no programs included more than 80% of the criteria. Most programs included 40%-60% of the criteria (Table 2). 

**Table 2:** Distribution of inclusion criteria on residency program websites

Percentage of criteria included (%)	Websites providing information (N = 272)
90-100	0
80-90	0
70-80	10
60-70	47
50-60	91
40-50	62
30-40	40
20-30	12
10-20	6
0-10	4

Application information

These 10 criteria from the 58-point set were underrepresented, with an average prevalence of only 50.1% (Table 1). The most frequently mentioned criteria included information regarding the application process (229/272, 84.2%), selection criteria (211/272, 77.6%), acceptance of international medical graduates (175/272, 64.3%), link to Electronic Residency Application Service (169/272, 62.1%), and contact email (159/272, 58.5%). On the other hand, the least commonly mentioned criteria included the interview process (106/272, 39.0%), interview dates (96/272, 35.3%), audition/visiting rotations (87/272, 32.0%), minimum United States Medical Licensing Examination scores (70/272, 25.7%), and mailing address (70/272, 25.7%).

Introduction

On average, the seven criteria within the "Introduction" section were referenced by 46.2% of the 272 residency programs (Table 1). The most frequently included were training sites (244/272, 89.7%), comprehensive faculty listing (232/272, 85.3%), and facility description (147/272, 54.0%). In contrast, the least commonly included were the chief resident’s message (6/272, 2.2%), chairman’s message (58/272, 21.3%), department changes and news (67/272, 24.6%), and program director’s message (133/272, 48.9%). 

Residency manual information

OB/GYN residency program websites incorporated the most criteria from this category (Table 1). Of the 58 total criteria, 12 belonged to this category, with an average prevalence of 59.5%. The criteria included benefits (238/272, 87.5%), salary (229/272, 84.2%), vacation (227/272, 83.5%), and meal allowance (178/272, 65.4%), parking (156/272, 57.4%), resident wellness (149/272, 54.8%), information about the surrounding area (144/272, 52.7%), maternal leave mentioned (142/272, 52.2%), paternal leave mentioned (139/272, 51.1%), moonlighting (137/272, 50.4%), incentives (136/272, 50.0%), and socializing events for residents (68/272, 25.0%). 

Didactics and program information 

With an average of 40.7%, this category scored the lowest in terms of average criteria presence (Table 1). Out of the 58 criteria, 15 fell within this category. The most frequently mentioned criteria included the rotation schedule (200/272, 73.5%), an ultrasound component (192/272, 70.6%), description of the didactics (169/272, 62.1%), meetings/conferences (168/272, 61.8%), journal club (163/272, 59.9%), simulation labs (145/272, 53.3%), and call requirements (140/272, 51.5%). The least frequently mentioned criteria included information about the imaging equipment (16/272, 5.9%), imaging/procedural numbers (32/272, 11.8%), surgical case/responsibility progression (41/272, 15.1%), surgical statistics (52/272, 19.1%), international opportunities (59/272, 21.7%), educational resources (83/272, 30.5%), robotics (107/272, 39.3%), and responsibility progression (106/272, 39%). 

Research

Among the 60 websites that were analyzed for their content, four criteria in this category appeared in an average of 42.6% of residency programs (Table 1). The most commonly mentioned were research requirements (166/272, 61.0%) and support to present research (148/272, 54.4%). In contrast, research resources in the department (60/272, 22.1%) and active/past research projects (93/272, 34.2%) were the least commonly highlighted.

Current residents' information

Across all programs, this category had a prevalence of 48.2% and encompassed eight criteria (Table 1). This included the number of residents in the program (238/272, 87.5%), current residents' listing (237/272, 87.1%), residents' photos (217/272, 79.8%), residents' education (207/272, 76.1%), residents' hometown (66/272, 24.3%), residents' extracurricular activities (64/272, 23.5%), residents' academic interests (23/272, 8.5%), and residents' research interest (5/272, 1.8%). 

Alumni: graduate/post-residency life 

Of the residency programs, 43.6% offered information on graduate/post-residency life (Table 1). Among the 272 programs analyzed, 121 (44.5%) indicated that their graduates pursued fellowships, while 118 (43.4%) noted that their graduates pursued work in private practice.

## Discussion

The study highlights a gap in the comprehensiveness of OB/GYN residency program websites in the US. On average, only 48.6% of the evaluated criteria were met, suggesting that many programs lack important information to help prospective applicants make informed decisions [17]. With the growing reliance on digital platforms for residency applications, especially after COVID-19, residency programs must enhance their online presence to remain accessible and competitive. Strengthening website content not only supports applicants but also reflects a program’s commitment to communication and accessibility, helping attract top candidates and improve its academic standing.

Our analysis found that OB/GYN residency program websites included only 28 out of the 58 evaluated criteria on average. Residency manuals contained the most comprehensive information, while didactics and program descriptions were less complete. This discrepancy suggests that programs should focus on improving the clarity and completeness of their websites. 

Recent studies have evaluated website comprehensiveness for fellowship and residency programs in general OB/GYN [2,5,18] and urogynecology [3]. Jain et al. found that American urogynecology program websites met an average of 46.46% of the total criteria, while Canadian urogynecology program websites met only 27.40% [3]. In comparison, our study found an average of 48.6%. Furthermore, our study revealed that American OB/GYN program websites scored significantly higher than Canadian OB/GYN program websites in a similar evaluation [5]. Specifically, the category with the highest prevalence on American OB/GYN program websites was wellness, while the category with the lowest prevalence was clinical work. In comparison, the category with the highest prevalence on Canadian OB/GYN program websites was faculty information, while the lowest was clinical work [5]. 

A second study by Jain et al. assessed the completeness of Canadian OB/GYN residency program websites and found that, similar to American urogynecology fellowship programs [3], the highest-scoring subcategory was physician wellness [5]. The lowest-scoring subcategory was current fellows, while research and education scored the highest [5]. In comparing fellowship and residency websites, the study found that fellowship websites scored higher than residency websites, though the difference was not statistically significant [5]. A previous study by Sardana et al. evaluated Maternal-Fetal Medicine fellowship program websites in terms of the content and accessibility of information across three domains: program overview, application process, and education [2]. It recommended that while most websites included at least one means of communication, they should include at least two to increase the accessibility of information between applicants and program administrators [2]. The study also highlighted that all three areas needed improvement, especially in the application process and education categories [2]. These findings [2,3,5] contrast with ours, where American OB/GYN residency websites had the most content on residency manuals and the least on didactics and program details.

Another study evaluating 76 women’s breast imaging radiology fellowship programs across Canada and the US found that most websites lacked essential information [19]. On average, only 11 out of the 27 (40.0%) criteria were met [19]. The least prevalent category was incentives, particularly in terms of career placement following fellowship completion [19]. However, like other studies, most program websites included sufficient information about the application process [19]. This study also found that most American and Canadian women’s breast imaging radiology fellowship programs provided insufficient information for potential applicants [19]. 

Limitations

Our study has its share of limitations. First, the data collection period was limited to two months (February to March 2020). Therefore, this study cannot account for any updates made to the program websites after data collection. Second, the 58 study criteria outlined in Table 1 may not fully capture all factors that are important to prospective applicants, as research identifying the specific information they seek on program websites is limited. To address this, we included a representative selection of variables commonly found in similar studies. Additionally, equity, diversity, and inclusion (EDI) elements were not included in our analysis, as they were not part of the 58-point criteria used in this study. However, these factors are increasingly recognized as important in medical education.

Future directions

There is a lack of research identifying the most critical information for prospective applicants. Future studies should focus on the factors that are most significant to individuals during the application process for training programs. Additionally, research should examine how EDI-related elements are represented on program websites to enhance the comprehensiveness of such evaluations.

## Conclusions

Most American OB/GYN residency program websites lack essential information that may be important for applicants. By improving the comprehensiveness of their content, these websites could better represent the program to prospective applicants. Enhancing website content not only helps applicants make more informed decisions but also helps programs attract candidates who align with their mission and values. Given the ever-increasing reliance on digital information, addressing these content gaps is more important than ever. Additionally, standardizing website content across programs could ensure that all applicants consistently have access to the information they need. Comprehensive and accessible websites can therefore play a significant role in supporting applicants and building program appeal in an increasingly competitive environment.
